# Locoregional Surgery in Metastatic Breast Cancer: Do Concomitant Metabolic Aspects Have a Role on the Management and Prognosis in this Setting?

**DOI:** 10.3390/jpm10040227

**Published:** 2020-11-13

**Authors:** Maria Ida Amabile, Federico Frusone, Alessandro De Luca, Domenico Tripodi, Giovanni Imbimbo, Silvia Lai, Vito D’Andrea, Salvatore Sorrenti, Alessio Molfino

**Affiliations:** 1 Department of Surgical Sciences, Sapienza University of Rome, 00161 Rome, Italy; federico.frusone@gmail.com (F.F.); dr.aless.deluca@gmail.com (A.D.L.); domenico.tripodi@uniroma1.it (D.T.); vito.dandrea@uniroma1.it (V.D.); salvatore.sorrenti@uniroma1.it (S.S.); 2 Department of Translational and Precision Medicine, Sapienza University of Rome, 00185 Rome, Italy; imbimbo.1638090@studenti.uniroma1.it (G.I.); silvia.lai@uniroma1.it (S.L.); alessio.molfino@uniroma1.it (A.M.)

**Keywords:** metastatic breast cancer, breast surgery, immune system, metabolic derangements, precision medicine, integrated therapies

## Abstract

Although they cannot be considered curative, the new therapeutic integrated advances in metastatic breast cancer (MBC) have substantially improved patient outcomes. Traditionally, surgery was confined to palliation of symptomatic or ulcerating lumps. Data suggest, in some cases, a possible additive role for more aggressive locoregional surgical therapy in combination with systemic treatments in the metastatic setting, although a low level of evidence has been shown in terms of improvement in overall survival in MBC patients treated with surgery and medical treatment compared to medical treatment alone. In this light, tumor heterogeneity remains a challenge. To effectively reshape the therapeutic approach to MBC, careful consideration of who is a good candidate for locoregional resection is paramount. The patient’s global health condition, impacting on cancer progression and morbidity and their associated molecular targets, have to be considered in treatment decision-making. In particular, more recently, research has been focused on the role of metabolic derangements, including the presence of metabolic syndrome, which represent well-known conditions related to breast cancer recurrence and distant metastasis and are, therefore, involved in the prognosis. In the present article, we focus on locoregional surgical strategies in MBC and whether concomitant metabolic derangements may have a role in prognosis.

## 1. Introduction

The prevalence of metastatic breast cancer (MBC) is about 3–6% in the United States [1], affecting 15,000 women annually [2], and it is estimated that 3–8% of patients with newly diagnosed breast cancer have distant metastases as an initial presentation [3]. Metastatic disease is particularly common in undeveloped countries, where up to 25% of patients present at stage IV at first diagnosis [4]. Interestingly, the median overall survival rate of MBC patients has improved over the last years (from 13 months in 1985 to 33 months in 2016), as well as the 5-year survival rate (from 10% in 1985 to 27% in 2016) [5].

The main goal in MBC treatment is to prolong survival and to maintain or improve the quality of life of the patient [6]. To achieve this, a large palette of anticancer treatments is at hand for use in the adjuvant and metastatic settings. Current therapeutic options for MBC management include radiotherapy, systemic treatments (i.e., hormonal therapy, monoclonal antibodies, chemotherapy, small molecule signal transduction inhibitors, antibody–drug conjugates), surgical treatment [7], as well as nutritional and metabolic interventions [8]. Regarding antineoplastic treatments, the choice is often based on the immunohistochemical characteristics of the breast cancer, according to receptor status [7]. This is an example of modern precision medicine in cancer patients, which relies on identifying key biomarkers driving tumor progression, representing novel therapeutic approaches [7], including genomic sequencing, which may help in the selection of personalized treatment as well as in assessing treatment resistance [9,10].

Although surgical treatment has usually been reserved for the palliative care of symptomatic MBC, i.e., patients with large exophytic masses or ulcerating breast lumps, recent data suggest a possibly expanding role for more aggressive locoregional therapy in combination with systemic therapy [7,11]. Khan et al. analyzed the data from the National Cancer Database of resections of the primary tumor in patients with MBC [12] and documented an improvement in 3-year survival in MBC patients undergoing surgery compared to those who did not. Moreover, patients with negative surgical margins presented the best prognosis [12]. Recent studies, conducted on homogeneous cohorts of MBC patients, have confirmed an improved survival rate after resection of the primary tumor, identifying several variables associated with the response to surgical resection, including younger age, having a single metastatic site, chemotherapy as first-line treatment, HER2-enriched tumor, and lower nodal burden [13,14]. Moreover, Rao et al. reported that MBC patients who had undergone breast surgery and the appropriate extent of axillary surgery had improved outcomes in terms of overall survival compared with patients who only had resection of the primary tumor and/or limited axillary surgery [15]. In this light, several clinical studies were conducted in the past few years to clarify the impact and role of locoregional surgical treatment in patients affected by MBC.

Moreover, it is clear that the treatment of MBC is rapidly evolving, driven by either a greater understanding of the biologic pathways underlying tumorigenesis and metastatic growth or the concept that immune surveillance supports and provides molecular mechanisms during tumor progression. A reduction of primary tumor volume determines a reduction of circulating tumor cells, and the role of the immune system has been hypothesized in promoting/suppressing metastatic growth [16].

An emerging clinically relevant aspect in the management of breast cancer is represented by metabolic and nutritional derangements before, during, and after anticancer therapies [8,17]. The majority of the data in the literature are available on specific risk factors (i.e., overweight/obesity, insulin-resistance) for tumorigenesis and cancer relapse [8].

However, the clinical management of metabolic derangements in MBC does not represent consolidated clinical practice, despite the available experimental and clinical evidence indicating their roles in negatively impacting the prognosis in the MBC setting.

In this light, in the present article, we focus on locoregional surgical strategies in MBC and whether concomitant metabolic derangements may have a role in clinical outcomes.

## 2. Breast Surgery in MBC: Where Are We Now?

### 2.1. Data from Retrospective Studies

Khan et al., in 2002, conducted a large retrospective study on more than 16,000 patients from the National Cancer Database and documented that women with MBC treated with locoregional treatment (mastectomy or local excision, both with R0 margins) had a better prognosis compared to patients with involved margins after locoregional surgery or who had not undergone surgical treatment [12] (Table 1). Lang et al. [13], in their study, found a significantly higher overall survival rate and progression-free survival in MBC patients who had undergone locoregional treatment when compared to patients who had not undergone surgery. The median survival of patients treated with surgery was 56.1 months compared to 37.1 months in patients who did not undergo surgical treatment. A higher overall survival was also associated with estrogen receptor positivity and having a single metastasis [13].

More recently, Vohra et al. considered 29,916 patients from the Surveillance, Epidemiology, and End Result program (SEER) database and found that MBC patients who had undergone primary tumor resection had a better median disease-specific survival compared to MBC patients who had not undergone locoregional treatment (34 versus 18 months) [18]. Other factors associated with better disease-specific survival were younger age, lower T and N stage, lower grade, luminal tumors, lower tumor grading, adjuvant radiotherapy, and surgery performed in the latter years [18], although no information was given on nutritional and metabolic status.

Lane et al. [11] presented, in 2019, the largest contemporary analysis to evaluate surgical resection of the primary tumor among women with MBC and its association with overall survival. The authors considered 24,015 stage IV breast cancer patients and found a survival improvement of patients who were undergone locoregional treatment, independent of treatment sequence. In fact, they had a median overall survival of 52.8 months in patients subjected to surgery after chemotherapy and a median overall survival of 49.4 months in patients subjected to surgical treatment before chemotherapy, compared to a median overall survival of 37.5 months in patients who underwent systemic treatment without surgery [11] (Table 1). Although these data suggest a benefit from surgery, it has to be considered that some patients may not be candidate for surgery, according to medical comorbidities or extension of the locoregional disease.

In the effort to further control for selection bias, the authors conducted an additional sensitivity subanalysis, considering only MBC patients in whom a diagnosis of clinical M1 disease and confirmation of known sites of metastatic disease were present, and this approach confirmed the initial results [11]. However, this is a retrospective study, which may limit the interpretation of the results obtained, and, to address these questions, some authors performed prospective randomized clinical trials. In the last few years, several randomized trials have investigated the role of locoregional treatment in stage IV breast cancer patients (Table 1).

### 2.2. Data from Prospective Studies

In 2015, Badwe et al. [19] conducted a randomized controlled trial on 350 patients with newly diagnosed MBC, who had responded to first-line chemotherapy, assigning them to two arms (locoregional treatment versus no-locoregional treatment). With a median follow-up of 23 months (IQR 12.2–38.7), the authors did not find significant differences in the two groups and thus no benefit of locoregional treatment. Moreover, the 2-year overall survival was 41.9% in the locoregional treatment and 43% in the no-locoregional treatment, and, furthermore, only 18% of patients who had undergone locoregional treatment required palliative surgery [19]. Finally, they found a reduction of progression-free survival in the group that had undergone locoregional treatment, hypothesizing that this was determined by the growth of the metastatic tumor as a result of the removal of the primary tumor, as showed by other preclinical studies [20,21,22,23]. The authors concluded that they did not find any evidence to support the use of surgical locoregional treatment to improve overall survival in MBC patients who responded to first-line chemotherapy and suggested not to consider this procedure in routine practice.

Conversely, Soran et al. later described results obtained by the MF07-1 trial, a multicenter, phase 3, randomized, controlled study that compared the locoregional treatment followed by systemic therapy with systemic therapy alone for newly diagnosed MBC patients [24]. The authors enrolled 274 patients and, despite the results documented by Badwe et al. [19], found that patients who had undergone locoregional treatment had a 34% lower hazard of death compared to systemic treatment alone, with a median follow-up of 54.5 months and 55 months, respectively. In particular, the survival rates were similar at 3 years (60% in the locoregional arm and 51% in the systemic therapy arm), but at 5 years, the percentage of alive patients was higher in the locoregional group (41.6% versus 24.4% of the systemic group) [24]. This is the first randomized study showing a significant improvement in the survival rate in patients with MBC treated with locoregional surgery, 5 years after treatment [24]. Analyzing the two groups, the authors found that particular subgroups of MBC patients were associated with higher overall survival after surgery, in particular, mainly luminal tumors, age <55 years, and solitary bone metastases. In this light, in patients with MBC, locoregional treatment might be an option to consider in a multidisciplinary setting according to age, performance status, tumor type, comorbidities, and metastatic tumor burden [24].

In particular, a debate exists due to the significant bias identified in these studies: (i) surgery may be a surrogate for more aggressive multimodal therapy, (ii) stage IV breast cancer patients may include women diagnosed either early by modern imaging or shortly after surgery, and (iii) MBC patients in better general condition are offered surgery, while patients with worse general status (i.e., presence of comorbidities, more frail) are not.

### 2.3. Data from the Cochrane Database and Ongoing Trials

A recent Cochrane systematic review [25] analyzed data on the effectiveness of breast surgery associated with medical treatment with respect to medical treatment alone in MBC patients. The authors have considered randomized clinical trials for the analyses, finally collecting only two studies involving a total of 624 women. The results did not show a clear improvement in survival in MBC patients treated with surgery and medical treatment compared to medical treatment alone, highlighting how the results were limited by a very low quality of evidence [25]. Further randomized clinical trials are needed to achieve more robust evidence and to better understand how the complex heterogeneity influences the prognosis.

In particular, in 2010, recruitment was initiated for the Eastern Cooperative Oncology Group (ECOG) E2108 randomized trial (https://clinicaltrials.gov/ct2/show/NCT01242800), including patients presenting with stage IV breast cancer. This is a 2-arm study (standard palliative therapy versus locoregional surgery on primary tumor), having as the primary end-point to determine if early locoregional surgical therapy improves overall survival and, as secondary end-points, to study the quality of life and control of chest wall disease. The results will potentially clarify these aspects and possibly change the management of patients with stage IV breast cancer disease.

## 3. Are There Other Factors Affecting the Choice for the Resection of the Primary Tumor in MBC?

Metastatic breast tumor management remains a challenge for physicians, and there is debate on the evidence that suggests that locoregional treatment of the primary tumor confers an overall survival advantage in this setting. Stage IV breast cancer represents a disease characterized by tremendous heterogeneity, as described by Lim and Hortobagyi [1]. In particular, differences in the underlying health status, i.e., age, comorbidities, performance status, and organ function, contribute to MBC presentation, affecting treatment decisions and patient outcomes [1].

There are gaps in the knowledge that may impact the decision-making process regarding who is a good candidate for locoregional resection in MBC. In this light, what risk factors need to be identified and thus treated to improve the prognosis of MBC patients remain unclear.

Treatment of MBC may target fundamentally different mechanisms than standard chemotherapeutic drugs, which are generally antiproliferative and, therefore, most efficiently eliminate rapidly growing cells.

Although a clinically apparent metastasis is usually associated with late stages of cancer development, micrometastatic dissemination may be an early phenomenon. Nonconclusive data are available on the molecular events, including changes in specific metabolic pathways underlying the development of metastatic disease, and this may impact the treatment’s decision process and, in part, may influence the response to surgical locoregional treatment [26,27,28].

First, the impact of the immune system on metastatic colonization is still unclear. Authors have theorized that disseminated tumor cells could metastasize, evading the immune system (actively, performing a sort of “immunoediting” or remaining “dormant”) [26,27]. Secondly, the destiny of disseminating tumor cells after the removal of the primary tumor is unclear. Despite the fact that retrospective clinical studies have demonstrated that complete resection of the primary tumor improves survival [11,18], experimental evidence has shown that ablation of the primary tumor accelerates the growth of disseminating tumor cells in metastatic sites [28,29], possibly due to systemic inflammatory response [30]. In contrast, in 2019, Piranlioglu et al. demonstrated in a mouse model that an innate and adaptive immune system, stimulated by the tumor (in particular CD8+ cells), may kill disseminating tumor cells after the complete resection of primary tumors, keeping an immunologic memory [16]. These results can be seen as a molecular explanation of improved overall survival in breast cancer patients, following primary tumor resections with clear margins.

In this light, the improvement in the survival rates of patients with MBC represents one of the major concerns in public health [1].

## 4. Emerging Metabolic Aspects: Do They Have a Key Role in MBC Management?

As previously shown, surgery in MBC represents a clinically relevant issue due to the controversial results obtained in different studies in terms of prognosis. In fact, some questions remain unanswered: (i) who is a target candidate for locoregional surgical during MBC? (ii) what are the risk factors related to MBC prognosis to be identified? (iii) Do metabolic changes affect the outcome(s) of MBC surgical procedure? (iv) Are specific metabolic interventions available in this setting?

We suggest that answers to these questions may derive from the implementation of precision (formerly called “personalized”) medicine. This can be defined as the possibility of managing a patient with the same taxonomic (affected by the same disease) disease differently to another by means of a tailored strategy based on strong evidence [31].

It is well known that there are different types of breast cancer, and it is a mixture rather than a single disease. Personalized medicine is based on tumor molecular profiles, and it is currently applied at different stages of breast cancer, including, especially, the prediction of treatment efficacy. One typical example of personalized medicine is represented by therapies implemented among patients with HER2-positive breast cancer compared to HER2-negative [32]. Moreover, a great challenge remains for the treatment of triple-negative breast cancer. This subtype, which is the most aggressive one, presents extensive and heterogeneous molecular features that need to be investigated in order to develop combined targeted agents to improve the efficacy of the treatment and possibly reduce disease progression. [33]. Although different tailored strategies have already been developed in the management of breast cancer patients, a paucity of data is available on MBC to obtain guidelines on a tailored therapeutic strategy. The specific and complex pathophysiology of MBC and its relationship with metabolic aspects should be considered to build new tailored approaches. In particular, the growing interest in metabolic derangements is emphasized by the role of altered glucose metabolism in driving the response to cancer treatment, its role in therapy resistance, and in cancer progression and metastasis [34].

Breast cancer metastasis is the systemic dissemination and colonization of cancer cells from the primary tumor to a secondary site and represents a major cause of cancer-related deaths [35]. The event of a circulating breast tumor cell, forming a metastatic colony in a distant organ, is extremely low [36]. Most cells that leave the tumor often die because of the inability to infiltrate distant organs. However, once metastasis occurs, breast cancer becomes a systemic disease, and, as previously indicated, the survival rate at 5 years decreases to 20% [36]. The heterogeneity between patients influences the journey of the cancer disease, as well as the prognosis and the treatment decisions [1]. The patient’s health conditions, which impact cancer progression and morbidity and their associated molecular targets, have to be considered for the treatment decisions and therapy development. Based on this, the metabolic syndrome represents a well-known condition related to breast cancer recurrence and distant metastasis and must, therefore, be accurately managed to improve the prognosis [37].

### 4.1. Metabolic Syndrome and Obesity

Metabolic syndrome is often associated with hormones and adipokines derangement, including changes in serum adiponectin, a polypeptide presenting properties related to glucose homeostasis and fatty acid oxidation [38,39]. In particular, adiponectin is involved in the pathogenesis of several obesity-related disorders and represents a potential therapeutic strategy for insulin resistance, type 2 diabetes, metabolic syndrome, and, more recently, carcinogenesis [40]. Clinical studies have linked obesity-related low adiponectin plasma levels with several types of cancer, including breast cancer [40,41,42], and with a more aggressive phenotype (i.e., larger size of tumor, high histological grade, and increased distant metastasis). In fact, in breast cancer, increased adiponectin levels may inhibit metastatic properties, including migration, adhesion, and invasion of cancer cells [43]. Accordingly, Taliaferro-Smith et al. have documented that adiponectin may block breast cancer cell invasion and migration, producing a profound modification in metastatic properties of breast cancer cells and thus presenting an antimetastatic effect [44].

There is significant epidemiologic evidence indicating that obesity promotes breast cancer development and progression [8] by secreting protumorigenic chemokines, growth factors, and fatty acids. However, the detailed mechanisms by which hypertrophic adipose tissue influences breast cancer cells are still not well understood. The peroxisome proliferator-activated receptors (PPARs) are ligand-activated transcription factors of the nuclear hormone receptor superfamily, regulating the expression of target genes involved in glucose and lipid metabolism and levels of inflammatory cytokines and adipokines. Data suggest that factors released by the adipose tissue may modify PPAR-regulated gene expression and lipid metabolism, inducing a more aggressive breast cancer cell phenotype. These effects are, at least in part, mediated by fatty acids provided by the adipose tissue [45].

Focusing on cancer-related risk factors associated with poor prognoses, such as obesity-related diseases and on their molecular pathways [8,45,46] (i.e., increasing adiponectin levels using adiponectin analogs, targeting specific PPAR-signaling), can potentially become an innovative personalized treatment for breast cancer patients and metastatic disease in improving the metabolic state and, therefore, response to systemic therapies, locoregional surgery, and overall survival.

### 4.2. Glucose Metabolism

Metabolic alterations in glucose metabolism in breast cancer are known to be associated with resistance towards conventional chemotherapy, and drugs modifying glucose metabolism have been identified to positively favor chemotherapy effects, possibly resensitizing the most aggressive breast cancer phenotypes, such as the triple-negative subtype, to novel treatments [34,47]. In this light, epidemiological studies showed that diabetic subjects on the metformin treatment regimen to control blood glucose levels had a lower risk of developing all type of cancers, and patients who were diabetic and on metformin treatment and were suffering from cancer, including breast cancer, had an improved response to chemotherapy, a better prognosis, and higher disease-free survival rates when compared to those who did not take metformin [47,48]. Metformin effects, which include inhibition of cell growth and proliferation-related pathways, as well as apoptotic cell death and reduction of tissue invasiveness and metastasis, may, in part, be related to the ability of metformin to reduce insulin resistance, insulin levels, and glucose circulation levels. In this light, adhering to an approach of precision medicine, including the treatment of well-known risk factors related to breast cancer recurrence and distant metastasis, may allow researchers to develop targeted combined therapies to improve the response to cancer therapies and prognosis.

### 4.3. MicroRNA Modulation

Interestingly, Farrè et al. have documented in experimental models that metabolic syndrome may influence the hyperactivation of C-terminal binding protein 1 (CTBP1), a corepressor of tumor suppressor genes, determining a crucial role in breast cancer progression through metastatic cascade activation (the regulation of multiple EMT-related genes and microRNAs) [37]. In this light, metabolic syndrome impacts breast cancer progression and the metastatic process, confirming that this condition has a key role to be considered in MBC patient’s prognosis and management [37].

Moreover, in this study, the authors analyzed the effect of metabolic syndrome and CTBP1 on miRNA regulation, showing that CTBP1 modulated several microRNAs implicated in cell proliferation and tumor progression [37]. MicroRNAs are noncoding small RNA that can negatively modulate gene expression, and they were recently considered either for their biological role and for their potential in the diagnosis and treatment of breast cancer [49].

In particular, the expression of miR-381-5p was detected as reduced in breast cancer tissue, and it was able to suppress cell migration and invasion [50]. Metabolic syndrome and CTBP1 were able to modulate miR-381-5p levels in xenografts generated in mice, and, in particular, CTBP1 promoted cell adhesion and migration by miR-181-5 repression [37]. In this light, microRNA profiling represents a promising approach in the integrated management of breast cancer.

## 5. Conclusions

In breast cancer, the identification of the most appropriate therapeutic strategies and their implementation in clinical practice appear challenging in the management of metastatic breast malignancies. However, the data available appear promising in MBC, although some are preliminary or obtained in experimental models. Regarding the surgical aspect, studies are not conclusive as to the improved survival rates in MBC patients undergoing resection of the primary tumor with clear margins. Interestingly, the analysis of the metabolic and clinical phenotypes—including modulation of adipokines (i.e., adiponectin) and miRNAs regulating metabolism—underlying the development of metastatic disease, which remains the principal cause of breast cancer-related deaths, may lead to the identification of more effective targeted approaches to prevent and treat metastases. According to the implementation of novel personalized treatments, surgical and metabolic strategies, when synergic, appear to be a promising, targeted, and integrated treatment approach to breast cancer. Extensive clinical evidence is expected to clarify these important aspects of MBC.

## Figures and Tables

**Table 1:** Studies considered in the present article that were conducted to investigate the impact of locoregional treatment compared to systemic therapy in MBC on prognosis.

Author (Year)	*N°* Patients	Time Period	Surgery	Outcome: Mortality *	PMID
**Khan (2002)**	16023	1990–1993	57.2%	HR 0.61 [95% CI 0.58–0.65] better prognosis	12407345
**Rapiti (2006)**	300	1977–1996	42%	HR 0.6 [95% CI 0.4–1] reduced risk of death	16702580
**Fields (2007)**	409	1996–2005	46%	aHR 0.53 [95% CI 0.42–0.67] reduced risk of death	17687611
**Gnerlich (2007)**	9734	1988–2003	47%	aHR 0.63 [95% CI 0.60–0.66] reduced risk of death	17522944
**Blanchard (2008)**	395	1973–1991	61%	HR 0.71 [95% CI 0.56–0.91] reduced risk of death	18438108
**Cady (2008)**	622	1970–2002	38%	Increased survival (*p* < 0.0001)	18726129
**Bafford (2009)**	147	1998–2005	41%	HR 0.47 (*p* = 0.003) reduced risk of death	18581232
**Le Scodan (2009)**	581	1980–2004	55%	HR 0.70 [95% CI 0.58–0.85] reduced risk of death	19204198
**Ruiterkamp (2009)**	728	1993–2004	40%	HR 0.62 [95% CI 0.51–0.76] reduced risk of death	19398188
**Khadakban (2013)**	196	2004–2009	25%	[95% CI 16.69–36.57] reduced risk of death	24426700
**Lang (2013)**	208	1997–2002	35.6%	HR 0.58 [95% CI 0.35–0.98] reduced risk of death	23306905
**Akay (2014)**	172	1994–2009	46%	HR 0.9 [95% CI 0.2–0.6] reduced risk of death	24510381
**Vohra (2018)**	29916	1988–2011	51%	increased survival (*p* < 0.0001)	29498453
**Lane (2019)**	24015	2003–2012	43.8%	HR 0.56 [95% CI 0.52–0.61] reduced risk of death	29227346
**Badwe (2015)**	350	2005–2013	50%	HR 1.04 [95% CI 0.81–1.34] no improvement in overall survival	26363985
**Soran (2018)**	274	2007–2012	50%	HR 0.66 [95% CI 0.49–0.88] reduced risk of death	29777404

* HR (hazard ratio) is indicated if available in the mentioned article.
